# The cost-effectiveness analysis of drug therapy versus surgery for symptomatic adenoid hypertrophy by a Markov model

**DOI:** 10.1007/s11136-019-02374-8

**Published:** 2019-11-28

**Authors:** Han Xiao, Jinqiang Huang, Weifeng Liu, Zihao Dai, Sui Peng, Zhenwei Peng, Ruiming Liang, Renqiang Ma, Yihui Wen, Jian Li, Weiping Wen

**Affiliations:** 1 Division of Medical Ultrasonics, The First Affiliated Hospital of Sun Yat-sen University, Guangzhou, China; 2 Department of Otolaryngology, The First Affiliated Hospital of Sun Yat-sen University, Guangzhou, China; 3 Department of Anesthesiology, The First Affiliated Hospital of Sun Yat-sen University, Guangzhou, China; 4 Department of Liver Surgery, The First Affiliated Hospital of Sun Yat-sen University, Guangzhou, China; 5 Clinical Trials Unit, The First Affiliated Hospital of Sun Yat-sen University, Guangzhou, China

**Keywords:** Adenoid hypertrophy, Markov model, Cost-effectiveness analysis, Adenoid surgery

## Abstract

**Purpose:**

Adenoid hypertrophy (AH) is common among young children. Adenoid-based surgery and drug therapy could be applied for symptomatic AH patients, yet the treatment decision is difficult to make due to the diverse cost and efficacy between these two treatments.

**Methods:**

A Markov simulation model was designed to estimate the cost-effectiveness (CE) of the adenoid-based surgery and the drug therapy for symptomatic AH patients. Transition probabilities, costs and utilities were extracted from early researches and expert opinions. Simulations using two set of parameter inputs for China and the USA were performed. Primary outcome was cost per QALY gained over a 6-year period. Deterministic and probabilistic sensitivity analyses were also conducted.

**Results:**

The utility for the surgery group and the drug group were 4.10 quality-adjusted life years (QALYs) and 3.58 QALYs, respectively. The cost of the surgery group was more than that of the drug group using model parameters specific to China ($1069.0 vs. $753.7) but was less for the USA ($1994.4 vs. $3977.7). Surgery was dominant over drug therapy when US specific parameters were used. Surgery group had an ICER of $604.0 per QALY when parameters specific to China was used.

**Conclusion:**

Surgery is cost-effective in the simulations for both China and the USA at WTP thresholds of $9633.1 and $62,517.5, respectively.

**Electronic supplementary material:**

The online version of this article (10.1007/s11136-019-02374-8) contains supplementary material, which is available to authorized users.

## Introduction

Adenoid plays an essential role in local and systemic immunity as well as the development of immune system in children [1]. Adenoid hypertrophy (AH) is common in children from 4 to 10 years old [2]. AH without any complications barely needs management. However, AH could result in symptoms like obstructive sleep apnea (OSA), rhinosinusitis, otitis media with effusion (OME) and recurrent upper respiratory tract infection [3], which could significantly affect the quality of life of children and lead to the need for medical interventions. For these patients, two treatment options are clinically considered. The first option is adenoid-based surgery, including the adenoid surgery, adenotonsillar surgery with or without otitis media surgery [2, 4]. Surgery could provide long-lasting improvement for quality of life, but is relatively invasive and expensive. Therefore, surgery is not routinely performed for each patient with various symptoms. To date, no consensus on the indications of the adenoid-based surgery has been established [4]. The second option is drug therapy, which is often cheaper than surgery but may be associated with a comparatively higher rate of symptomatic relapse [3, 5–7]. While the cost and efficacy are both quite different between the two strategies, the cost-effectiveness (CE) between the surgery and the drug therapy needs to be evaluated. While several studies evaluating the effectiveness of adenoid-based surgery and drug therapy for the management of symptomatic AH have been published [8, 9], the evaluation period in these studies is short [may choose to provide a range]. Furthermore, the cost and effectiveness of adenoid-based surgery and drug therapy should be jointly considered to inform decisions around the proper management of symptomatic AH [10]. To date, no cost-effectiveness study comparing adenoid-based surgery versus drug therapy over a longer-term period has been published. The aim of this study is therefore to assess the cost-effectiveness of adenoid-based surgery versus drug therapy for the management of symptomatic AH in 4-year-olds over a 6-year period. Yet cost and effectiveness vary among different areas. Here in this article, we choose the USA, a western developed country, and China, an eastern developing country to compare the cost-effectiveness between different treatments as well as between different areas.

The Markov models have advantages in this situation. Generally, the Markov model is applied in describing stochastic process, which is a random process that evolves over time [11]. By dividing the natural pathway of a disease, assigning transitions probabilities for movements between different pathways, and estimating the total costs and health benefits after the evolution of the disease, the CE of the intervention can be analyzed [11]. The Markov model has already been widely adopted in the evaluation of disease screening or treatments around the world [12–14]. The advantage of Markov model by taking both costs and outcomes over a period of time into accounts makes it particularly suited to modeling the progression of chronic disease. Especially, the cost, benefits and transitions probabilities could be set as a range in the analysis. Therefore, all circumstances within these ranges could be simulated during analysis so that costs and efficacies of different symptoms could be analyzed in one model.

Based on the Markov model, we analyzed the outcome of the disease and the CE of the different treatments, aimed to provide some evidences for the treatment for symptomatic AH patients.

## Methods

### Model construction

**Fig. 1:**
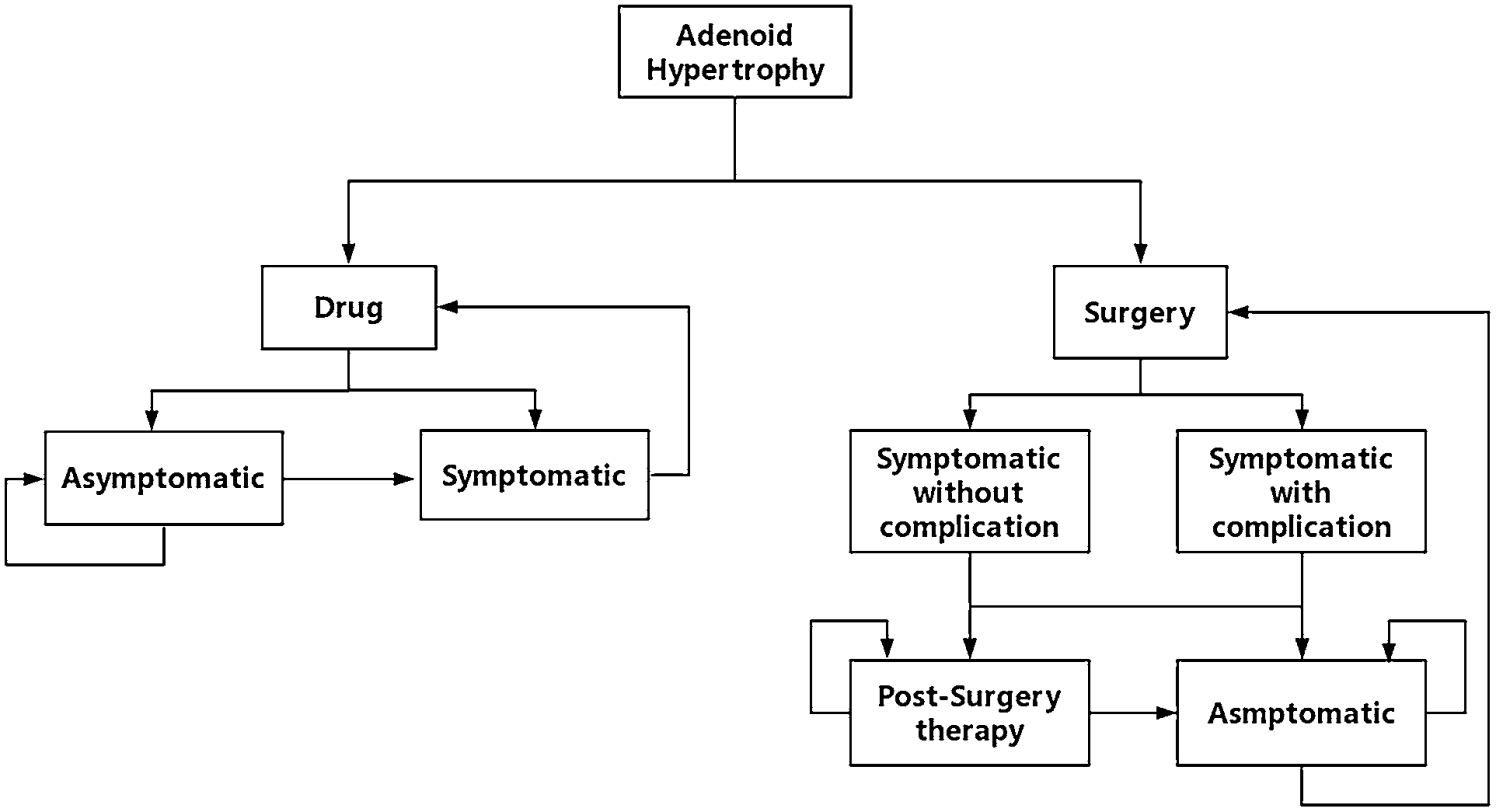
Flow diagram of the Markov cohort model. Two groups were divided as the drug group and the surgery group. Straight lines with arrows between the panes represent the process of patients’ health condition during the treatments

A Markov simulation model was designed to estimate the CE of the drug therapy and the surgery in the treatment of symptomatic AH. The model consisted of two decision strategies including the adenoid surgery and the drug therapy. Since AH without any symptoms barely needs management [15], we focused on whether the patients were symptomatic or not which affected the CE [16]. Considering the drug therapy mainly lasts for 3 months [3, 7, 17], the cycle time was set to be 3 months. Since the epidemiological data showed that the mean age of the patients who underwent adenoid surgery was 4 while most of the patients was under 10 [2], and naturally after the age of 10 most adenoids will stop hypertrophy and begin to atrophy, we considered a 6-year time horizon was proper for this study. Four health states were derived in the surgery group: symptomatic for surgery with complications (SSwC), symptomatic for surgery without complications (SSnC), post-surgery therapy (PS) and asymptomatic after surgery (AS). At first, all patients were symptomatic and were divided into SSwC and SSnC according to whether there were complications in the surgery. Patients who were asymptomatic after surgery transferred into AS, and some of those symptomatic would take a course of post-drug therapy and transferred into PS. Patients in the state of PS would transfer into AS if they were asymptomatic after a 3-months post-surgery therapy, or else they would stay in the state of PS and go on with another course of post-surgery therapy. Patients in the AS state will stay in this stage until the symptomatic AH relapse, when the patients will take another surgery and transfer into SSwC and SSnC again. Two health states were derived in the drug group: symptomatic and asymptomatic. All patients were in symptomatic state at first and would transfer to asymptomatic state or stay the same according to whether the drug therapy was effective. Patients asymptomatic after treatment were assumed to receive no further active treatment. The flow diagram of this model is shown in Fig. 1. Two sets of parameter inputs were used to model the experience of people with symptomatic AH in China and the USA. The mortality risk was not considered in this model since death in this population is extremely rare.

### Literature review

Transition probabilities, utilities and costs (Table 1) were derived from the literature identified through PubMed and Cochrane Library database with the latest searching on March 18, 2017. The medical subject heading (MESH) “Adenoid hypertrophy,” “adenoidectomy,” “adenotonsillectomy,” “the adenoid surgery,” “adenoid rhinosinusitis,” “adenoid steroids,” “adenoid otitis media with effusion,” “adenoid medication” and “costs and cost analysis” were applied. Reference lists of the included studies were hand-searched to identify further relevant articles.

**Table 1:** The transition probabilities, utilities and costs in the Markov model

Model parameter	Description	China mean (range)	USA mean (range)
Transitions			
Surgery			
Sur Comp	Probability of complication after treatment	0.024 (0.023 to 0.035)	
Sur Asym	Probability of no symptom after treatment	0.728 (0.469 to 0.967)	
Sur Re	Probability of recurrence after treatment	0.429 (0.429 to 7.400^a^)	
Drug therapy			
Drug Sym	Probability of symptoms after treatment	0.372 (0.233 to 0.500)	
Drug Re	Probability of recurrence after treatment	0.244 (0.200 to 0.268)	
Costs			
Surgery			
Cost Surg	Cost of surgery	$1000 ($527 to $1196)	$1658 ($1093 to $2866)
Cost Comp	Cost of surgery and cost of complication	$319 ($287 to $351)	$1605 ($883 to $3226)
Drug			
Drug cost	Cost of the drug therapy	$130.5 ($130.5 to $145)	$688.5 ($567 to $3888)
Utilities			
Eff Sym	Health state utility of the symptomatic patients	0.778	0.611–0.81
Eff Sur	Health state utility of the asymptomatic patients in the surgery group	0.854	0.828–0.882
Eff Drug	Health state utility of the asymptomatic patients in the drug group	0.965	0.89–0.965

^a^× 10^−3^

### Transition probabilities

For the surgery group, in order to fit all kinds of circumstances, we set the range of the probabilities with all types of adenoid-based surgeries in the sensitivity analysis, and an average rate was calculated for the Markov cohort analysis. The transition probabilities for one cycle were estimated from the original data using the declining exponential approximation of life expectance (DEALE) equation. An effective surgery was defined as all symptoms caused by AH were relieved after surgery. The average effective rate of the surgery was 72.8% [4, 18–29]. We assumed that those remained symptomatic after surgery would take one or more courses of post-surgery therapy. Post-surgery therapy was defined as a course of drug therapy that was similar to the drug group which would last until the symptoms were relieved. Symptom relapse rate was used instead of the AH relapse rate, since that it was the symptoms instead of the adenoid tissue that required another round of treatment. The probability of symptomatic relapse was assumed to be the probability that asymptomatic patients would experience symptoms again after regeneration of adenoid tissue. The symptom relapse rate after the surgery was estimated to be 0.000429 (0.000429, 0.0074) [30]. The re-operation was deemed as the revision adenoidectomy [31]. The complications rates were small and close between various kinds of surgeries, which was estimated from several relevant clinical trials with larger sample size to be 0.024 (0.023, 0.035) [32–34]. For the cost, effectiveness and effective rate of the post-surgery therapy, we assumed it the same with the drug group described below.

The intranasal corticosteroids (ICS) and the oral montelukast (OM) are the main medications of drug therapy world widely. We assumed the continuous therapy as the drug therapy in our model according to reported clinical trials [7, 35]. Since both the ICS and the OM are anti-inflammatory drugs with a close effectiveness, we supposed them homogeneous and estimated the ineffective rate every 3 months to be 0.372 (0.233, 0.5) [3, 36, 37]. The symptom relapse rate after drug therapy was derived from a clinical trial and was estimated to be 0.244 (0.220, 0.268) [38]. For the patients with symptoms relapse, we assumed that they would continue the drug therapy to relieve the symptoms according to clinical experience. As for the complications, there was a meta-analysis [7] discussing the safety of the ICS, indicating that the complications were rare. And there were no severe complications reported in the drug therapy. In view of this, we assumed that the influence of complications of drug therapy was negligible.

The average rates were calculated by meta-analysis for data from multiple studies (the effective rate of the surgery, the complication rate of the surgery and ineffective rate of the drug therapy). The symptom relapse rate after the surgery was derived from two studies with quite different samples (one was 1411 patients, and the other was 59). Rate from the study with larger sample size was used as the base value, and rate from the other study was used as the range. Only one study reported the symptom relapse rate after drug therapy and was used as the base value. The range for the symptom relapse rate was set as 90–110% of the base value.

### Costs and utilities

The study was conducted from a perspective of health care system; therefore, only direct medical costs were included. Costs in China and in the USA are quite different in both surgery and drug therapy group. Therefore, different costs were set for the two models separately. The total costs for each cycle were estimated with the frequency and unit cost of drugs, procedures, inpatient and outpatient visits, laboratory testing and imaging examination, and all were converted to US dollars in 2018 using the Component of Consumer Price Index [39, 40]. The median cost of the adenoid surgery was USD $1658.4 for the USA [25, 41–43] and $1000.4 for China [24]. Cost of the complications was USD $1604.6 for the USA [44] and $318.9 for China [44, 45]. Costs for the drug group were extracted and calculated according to researches for the USA [3, 41, 42] and according to experts’ opinions in China due to limited data. The utilities in each health state were extracted from literature [46–52] (Table 1), with data from different countries involved, and these extracted values did not vary a lot in different countries. All of the values accounted for the range of costs and utilities, and the medians were used as the base value. A discount rate was set as 3% yearly for both costs and utilities. Half cycle correction was implemented in this model.

### Cost-effectiveness analysis

Each health state had a corresponding cost and utility. Cost was defined as described above. The cost-effectiveness was measured using quality-adjusted life year (QALY), incremental cost-effectiveness ratio (ICER) and net monetary benefit (NB). QALY is a generic measure of disease burden, 1 QALY equates to one year in perfect health. QALYs are accrued at a rate of less than 1 per year for patients with symptoms. The ICER is the difference in cost between two possible interventions, divided by the difference in their effect. It represents the average incremental cost associated with 1 additional unit of the measure of effect, which related to 1 QALY in this study. NB, which takes the cost, effectiveness and willingness-to-pay (WTP) together into another single measurement, was used in the model to reduce the ambiguous implication caused by the mathematical uncertainty of ICER. The larger the NB is, the more cost-effective the treatment is. Tornado diagrams analyzing the sensitivity of each parameter in Markov model were performed. We aimed at simulating and analyzing the cost-effectiveness using model parameters specific to both China and the USA, and WTPs were set to be the per capita income in 2018 (USD $9633.1 for China and USD $62,517.5 for the USA) [53]. For the fixed WTP parameter, the strategy with the higher NB is more cost-effective. Roll-back analysis was performed to select a more cost-effective strategy. If the ICER of one strategy compared to another was less than WTP, the former strategy was considered as the more cost-effective one [13]. To evaluate the total impact of parameter uncertainties, the Monte Carlo probabilistic sensitivity analysis was performed with 10,000 simulations for each group, with the results showed diagrammatically in the form of a CE acceptability curve and a CE plane. While analyzing, the transition probabilities and utilities were considered to obey beta distribution, and the costs were considered to obey gamma distribution. The TreeAge–Pro–2008 software (TreeAge Software Inc., Williamstown, MA, USA) was applied to create a Markov model.

## Results

### Base-case analysis

After a 24-stage (6 years) cycling, a total of 99.9% of the patients in the surgery group were asymptomatic, compared to 72.0% in the drug group. As shown in Table 2, surgical management of symptomatic AH dominated drug management by being cheaper and more effective, using model parameters specific to the USA (incremental cost = − $1983.3; incremental effectiveness = + 0.52 QALYs). The ICER for the surgery group was $604 per QALY in China (incremental cost = $315.3; incremental effectiveness = + 0.52 QALYs).

**Table 2:** Cost-effectiveness between different treatment strategies

	China		US	
	Cost	QALYs	Cost	QALYs
Surgery	1069.0	4.10	1994.4	4.10
Drug	753.7	3.58	3977.7	3.58
Incremental cost/QALY	315.3	0.52	− 1983.3	0.52
ICER	604.0		Dominant	

Life years have not been presented because all-cause mortality is exogenous to the model

*ICER* incremental cost-effectiveness ratio; *QALY* quality-adjusted life years

### One-way and two-way sensitivity analysis

**Fig. 2:**
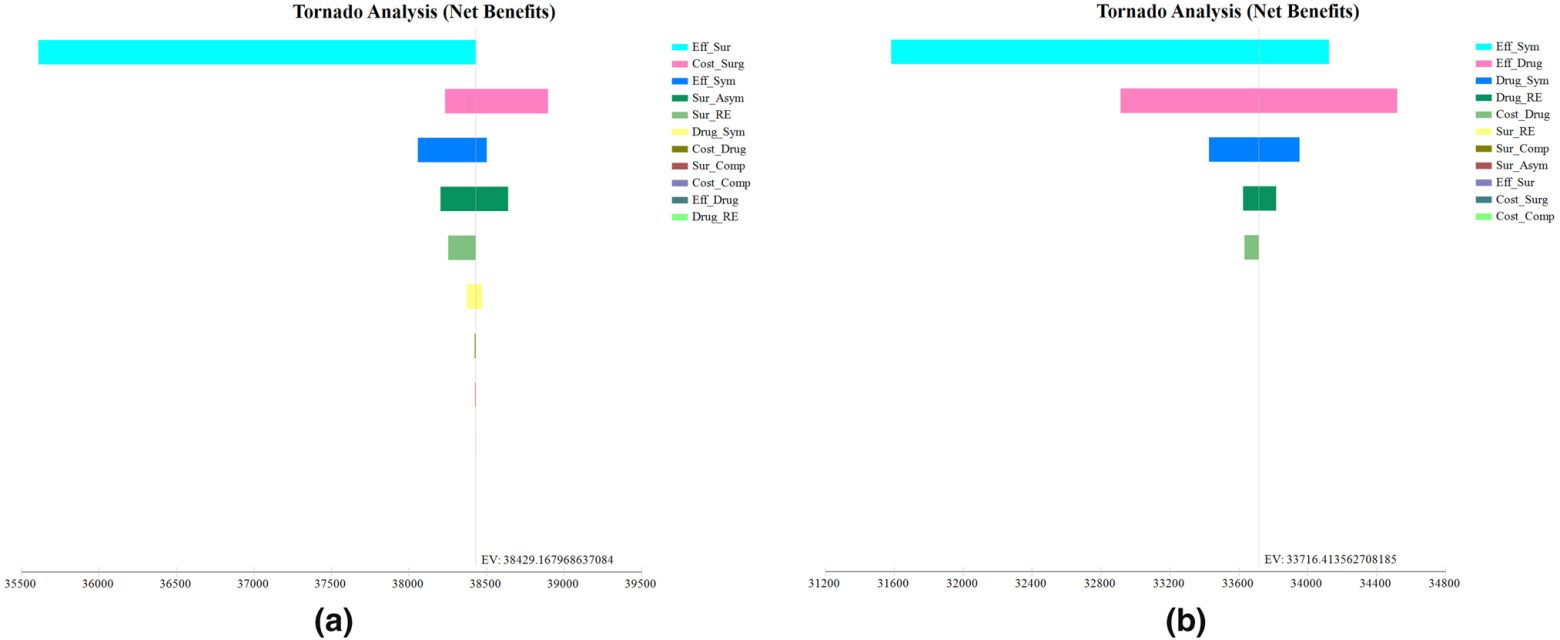
The tornado diagrams for China. The tornado diagrams are sensitivity analyses including all the parameters set in the model for the surgery group (**a**) and the drug group (**b**), respectively. The longer the bar is, the more influence the parameter has on the cost-effectiveness of the certain group. Abbreviations: health utility of a symptomatic state (Eff_Sym), health utility of asymptomatic patients in surgery group (Eff_Sur), health utility of asymptomatic patients in the drug group (Eff_Drug), cost of the surgery (Cost_Surg), cost of the surgical complications (Cost_Comp), cost of a 3-month drug therapy (Cost_Drug), the rate of surgery complications (Sur_Comp), the effective rate of the surgery (Sur_Asym), the ineffective rate of the drug therapy (Dru_Asym), the recurrence rate after surgery (Sur_RE), the recurrence rate after drug therapy (Dru_RE)

**Fig. 3:**
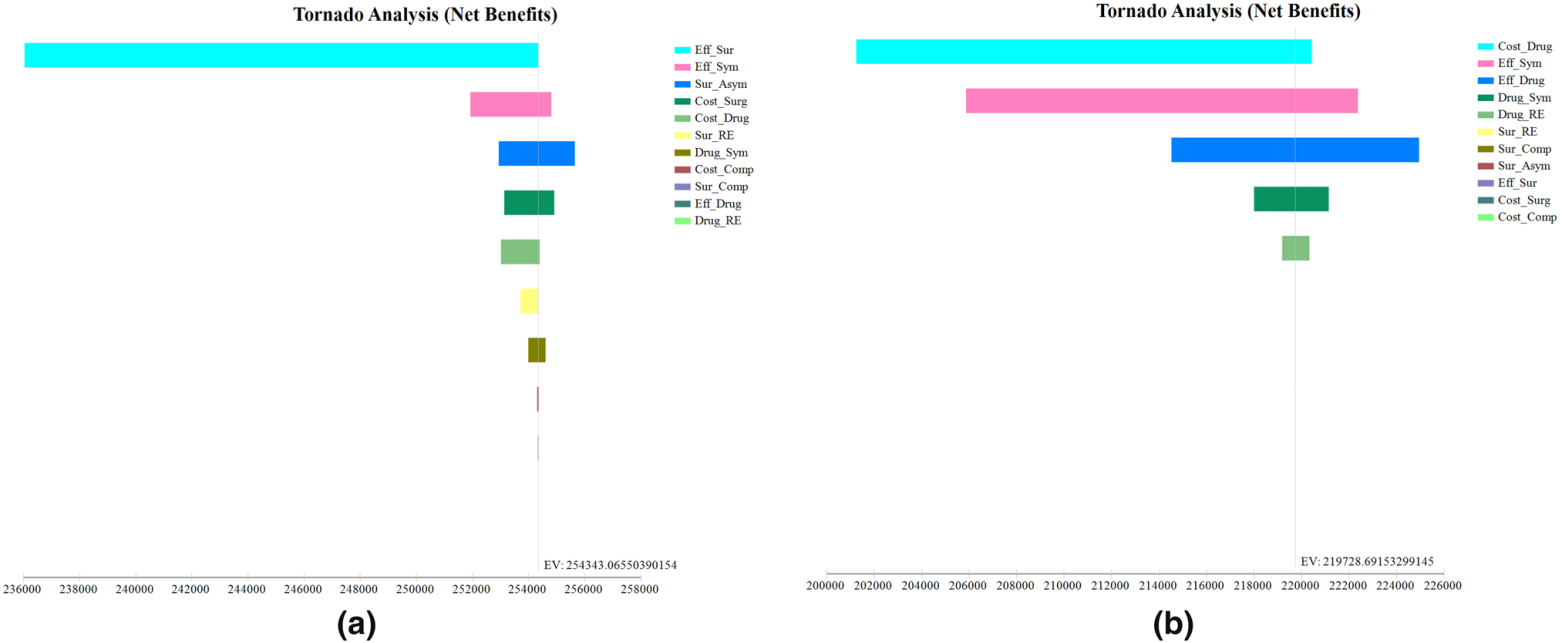
The tornado diagrams for the USA. The tornado diagrams are sensitivity analyses including all the parameters set in the model for the surgery group (**a**) and the drug group (**b**), respectively. The longer the bar is, the more influence the parameter has on the cost-effectiveness of the certain group. Abbreviations: health utility of a symptomatic state (Eff_Sym), health utility of asymptomatic patients in surgery group (Eff_Sur), health utility of asymptomatic patients in the drug group (Eff_Drug), cost of the surgery (Cost_Surg), cost of the surgical complications (Cost_Comp), cost of a 3-month drug therapy (Cost_Drug), the rate of surgery complications (Sur_Comp), the effective rate of the surgery (Sur_Asym), the ineffective rate of the drug therapy (Dru_Asym), the recurrence rate after surgery (Sur_RE), the recurrence rate after drug therapy (Dru_RE)

**Fig. 4:**
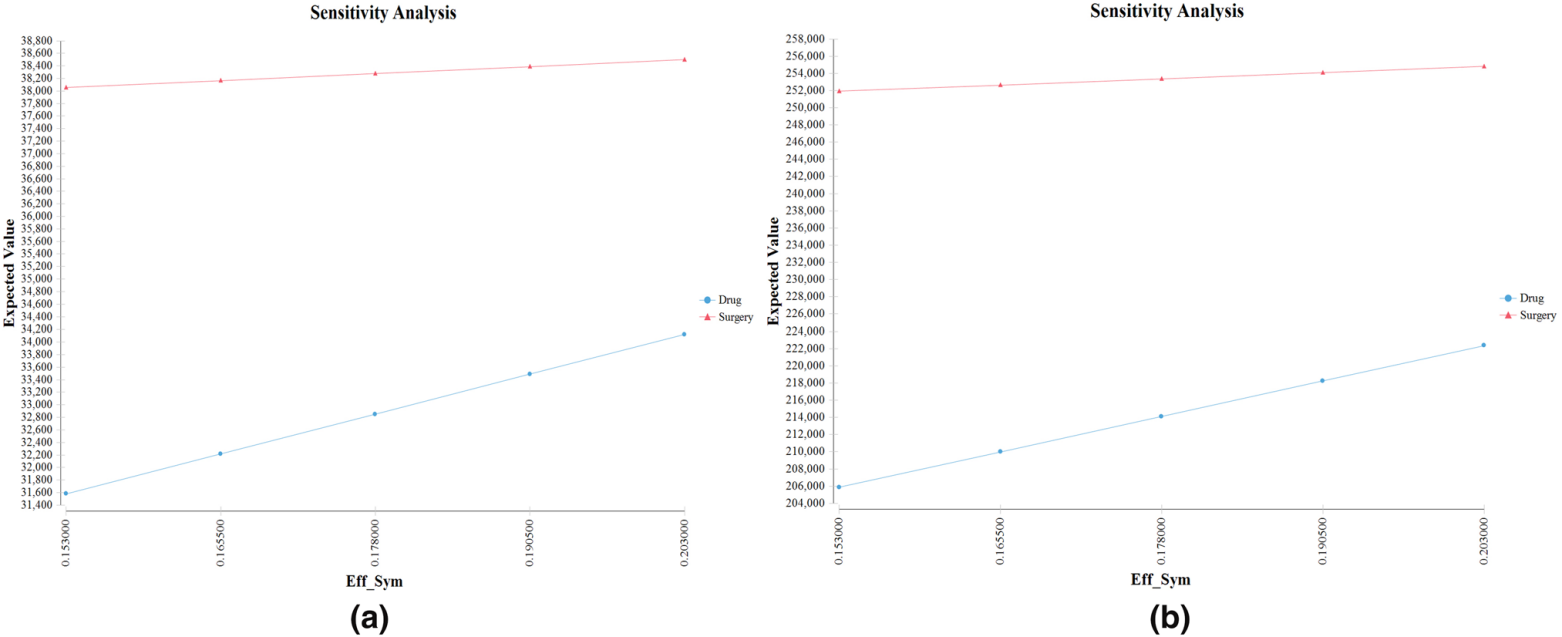
One-way sensitivity analysis of health utility of a symptomatic state. One-way sensitivity analysis of health utility of a symptomatic state for China (**a**) and the USA (**b**). The line above represents a better cost-effectiveness. Abbreviations: health utility of a symptomatic state (Eff_Sym)

In China, the three most sensitive parameters were the health state utility of the asymptomatic patients in the surgery group (Eff_Sur), the cost of the surgery (Cost_Sur), and health state utility of the symptomatic patients (Eff_Sym) for the surgery group, and were health state utility of the symptomatic patients (Eff_Sym), health state utility of the asymptomatic patients in the drug group (Eff_Drug) and the probability of the symptoms after drug therapy (Drug_Sym) for the drug group (Fig. 2). In the USA, the three most sensitive parameters were the health state utility of the asymptomatic patients in the surgery group (Eff_Sur), health state utility of the symptomatic patients (Eff_Sym) and the effective rate of the surgery (Sur_Asym) for the surgery group, and were the cost of a 3-month drug therapy (Cost_Drug), the health state utility of the symptomatic patients (Eff_Sym) and health state utility of the asymptomatic patients in the drug group (Eff_Drug) for the drug group (Fig. 3). Surgery group would always have a better cost-effectiveness in the one-way sensitivity analysis. Particularly, we analyzed the influence of the health utility of a symptomatic state to the cost-effective outcomes of both groups (Fig. 4), turned out that the lower the health utility of a symptomatic state was, the larger the gap of NB between surgery and drug therapy would be. In another word, a patient with severe symptoms would more likely to have an obviously better cost-effective outcome in the surgery group than in the drug group. A tornado diagram analyzing all the parameters was shown in Supplementary Fig. 1; the top two sensitive parameters for each model were further included in the two-way analysis. The two-way analysis demonstrated that, however, the two parameters change, surgery always had a better CE in both China and the USA.

### Monte Carlo probabilistic sensitivity analysis

**Fig. 5:**
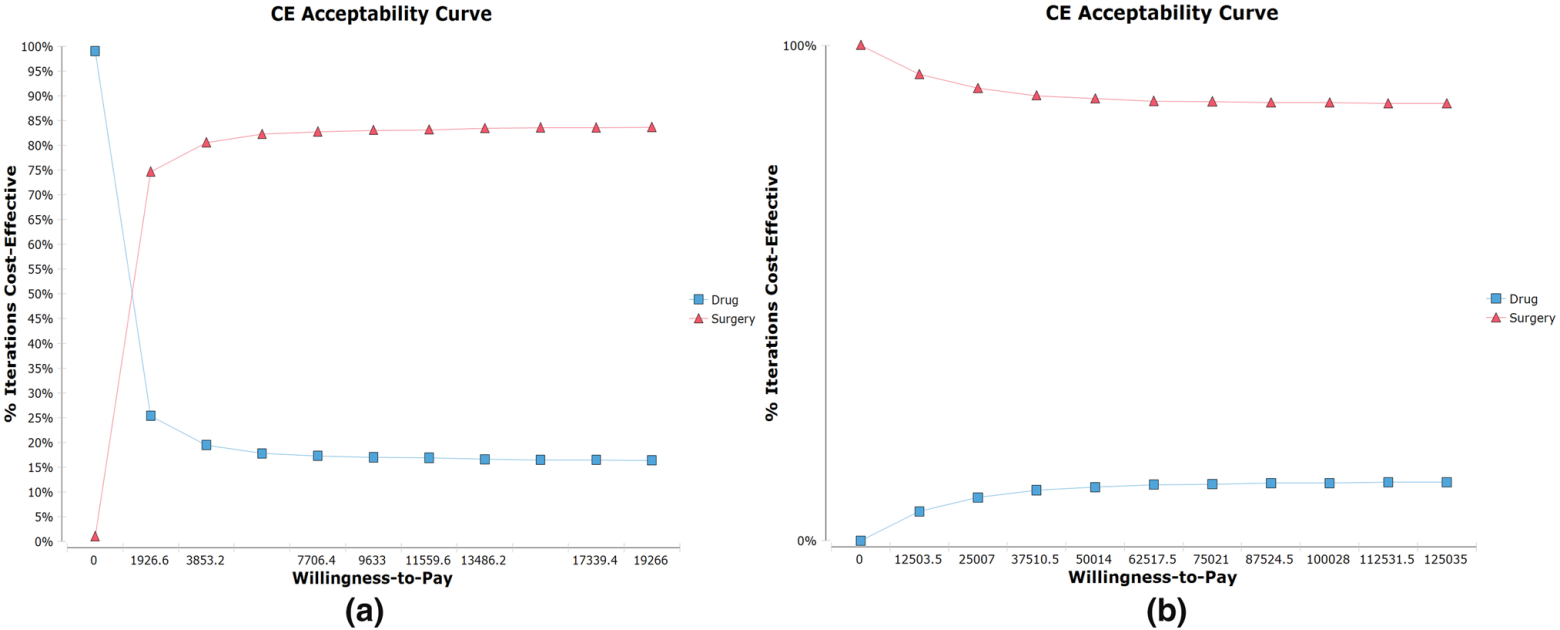
The cost-effectiveness acceptability curve of the two strategies. The cost-effectiveness acceptability curve for China (**a**) and the USA (**b**). The curve on the upside indicates a better cost-effective outcome for the population with a certain willingness-to-pay (WTP)

We also ran a Monte Carlo simulation with samples of 10,000 for both groups to analyze the probabilistic estimates. The simulation confirmed the conclusion of the base-case analysis that the surgery group was considered to be more effective and costly in the settings of China, but less costly in the settings of the USA (Supplementary Fig. 2). The probability that surgery was more cost-effective in China (WTP set to be $9633.1) and the USA (WTP set to be $62,517.5) was 0.847 and 0.887, respectively. We also calculated a CE acceptability curve to assess the probability that one therapy would be considered cost-effective for different threshold values of WTP (Fig. 5). With parameters specific to China, the surgery group was more likely to be cost-effective if WTP was more than $1518.3. With parameters specific to the USA, the surgery group was more cost-effective whatever the WTP was.

## Discussion

This study conducted a Markov model to compare the CE of surgery versus drug therapy for symptomatic AH patients. According to our results, surgery could achieve better efficacy with lower costs in a 6-year term. Drug therapy was more cost-effective in China only if the WTP was low. Surgery was more cost-effective in the USA under all circumstances. For patient with severe symptoms, surgery was likely to have a more dominate cost-effective outcome.

Currently, the adenoid-based surgery has become one of the most commonly performed pediatric surgical procedures worldwide [54]. In recent 20 years, the effectiveness and safety of surgery for adenoid hypertrophy have been greatly improved. New endoscopic technique and instruments help surgeons make resection of adenoid under direct monitor with less complications. However, the higher cost of surgery also brings doctors and patients trouble to make a decision. And with the effectiveness of the drug therapy rather unsatisfactory [7, 55, 56], the proper management for symptomatic AH patients is still unclear. Therefore, a cost-effectiveness analysis is necessary for deciding the proper treatment selection.

The comparisons of CE between surgery and drug therapy share a similar pattern in China and the USA. The surgery group has a better CE in the long term of treatment than the drug therapy according to the base-case analysis. We explain the result that the adenoid could obstruct the upper respiratory tract and the Eustachian tube, serve as a bacterial reservoir for infection and evoke the immune reaction [15, 57, 58], which could be removed with surgery once for all, while the incomplete remission of the drug therapy leads to a lower utility. Furthermore, the frequent recurrence of the symptoms requires another cycle of drug therapy increases the costs, making it less cost-effective [35, 38].

Results of cost-effectiveness acceptability were different between China and the USA, which we thought was mainly caused by the difference in the cost of drugs. The cost of drugs is much lower in China, reducing the gap of cost-effectiveness compared to surgery. For areas with WTP under $1518.3, drug therapy might be optimal. Besides, the sensitivity analysis (Fig. 5) showed that the lower the health utility of a symptomatic state was, the larger the advantage of a surgery therapy would be. This result can be a proof for the rationality of clinical situations that the doctors would recommend the surgery based on the adenoid size and the severity of the disease [59, 60].

There are some limitations for this research. First, most of the data were derived from cohort studies or reviews and from only several RCTs in each group. Therefore, we included as many studies as we can to control bias. Second, the effect on immunal functions and the changes of utility caused by surgical complications were not taken into the utility of the surgery group which could cause its advantages over drug therapy. However, several studies demonstrated that there were no negative short- or long-term impacts of the adenoid surgery [61, 62], and the surgical complications of AH surgery usually recovered within a few days. We assumed that the impact might not change the priority of surgery in cost-effectiveness. Third, the results of our model were based on studies that covered patients within the common age range for AH patients. The conclusions of this article may not apply to patients beyond this age range.

## Conclusion

The surgery is more cost-effective in the simulation of both China and the USA at WTP thresholds of $9633.1 and $62,517.5. Surgery is more cost-effective for those with severe symptoms.

## Electronic supplementary material

Below is the link to the electronic supplementary material.

Supplementary material 1 (DOC 2068 kb)
