# Supplementary material for: The cost-effectiveness analysis of drug therapy versus surgery for symptomatic adenoid hypertrophy by a Markov model
Source: Qual Life Res. 2019 Nov 28;29(3):629–38. doi: 10.1007/s11136-019-02374-8 (PMC7028839; doi:10.1007/s11136-019-02374-8)
Supplement: Supplementary file 1 — Supplementary material 1 (DOC 2068 kb) [file 11136_2019_2374_MOESM1_ESM.doc]

**The** [**Cost-effectiveness**](https://www.ncbi.nlm.nih.gov/pubmed/25188602) **analysis of Drug Therapy versus Surgery for** **Symptomatic Adenoid Hypertrophy by** [**a Markov Model.**](https://www.ncbi.nlm.nih.gov/pubmed/25188602)

Han Xiao^1※^, MD, Jinqiang Huang^2※^, MD, Weifeng Liu^3^, MD, Zihao Dai^4^, MD, Sui Peng^1,5^, MD, PhD, Zhenwei Peng^5^, MD, PhD, Renqiang Ma^2^, MD, PhD, Yihui Wen^2^, MD, PhD, Jian Li^2◎^, MD, PhD, Wei-Ping Wen^2◎^, MD, PhD

**◎ Corresponding Authors: Wei-Ping Wen, Jian Li, Department of** **Otolaryngology,** The First Affiliated Hospital of Sun-Yat Sen University.

Address: 58 ZhongshanEr Road, Guangzhou 510080, China.

Fax: 86-20-87333733

Tel:86-20-87333733

Wei-Ping Wen: [wenwp@mail.sysu.edu.cn](mailto:wenwp@mail.sysu.edu.cn); Jian Li: [lijianent@hotmail.com](mailto:lijianent@hotmail.com)

**Supplementary Figure 1. The tornado diagram including two strategies.**

**Supplementary Figure 2. The cost effectiveness scatterplot.**


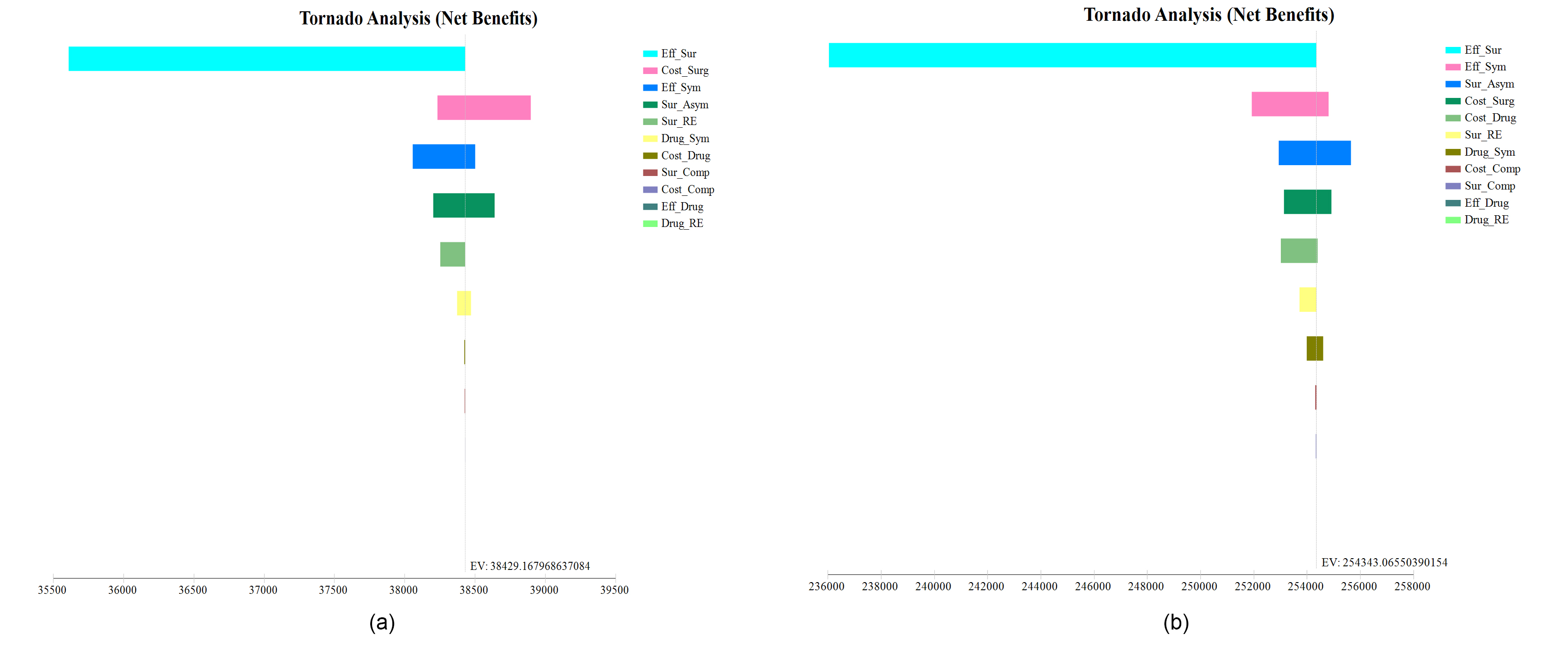


**Supplementary Figure 1. The tornado diagram including two strategies.** The tornado diagram analyzed the influence of all the parameters of the Markov model. The bar with a black mark on the left side indicates that this parameter would make a difference on the choose of dominant strategy while it varying in its range. Figure (a) stands for China and (b) stands for the United States. Abbreviations: health utility of a symptomatic state (Eff_Sym), health utility of asymptomatic patients in surgery group (Eff_Sur), health utility of asymptomatic patients in the drug group (Eff_Drug), cost of the surgery (Cost_Surg), cost of the surgical complications (Cost_Comp), cost of a 3-month drug therapy (Cost_Drug), the rate of surgery complications (Sur_Comp), the effective rate of the surgery (Sur_Asym), the ineffective rate of the drug therapy (Dru_Asym), the recurrence rate after surgery (Sur_RE), the recurrence rate after drug therapy (Dru_RE).


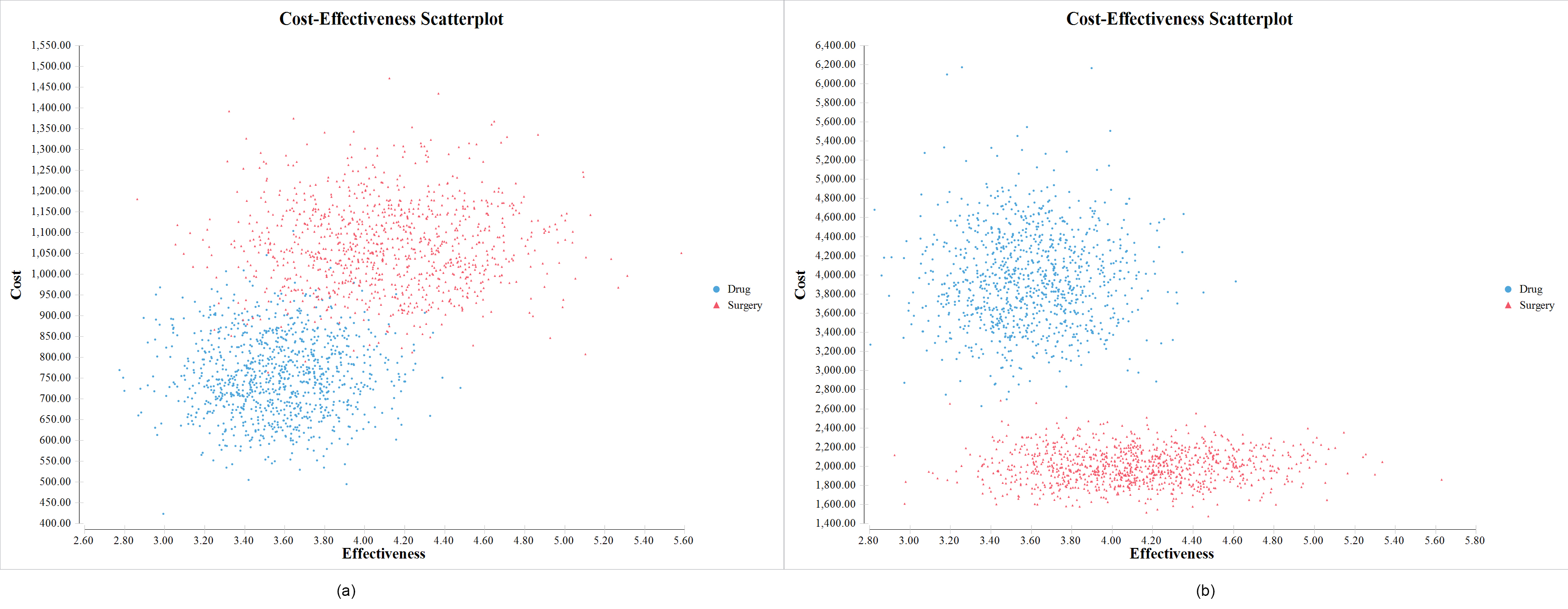


**Supplementary Figure 2. The cost effectiveness scatterplot.** Each point represents the result of one time of the Monte-Carlo simulation. The red points are for the surgery group while the blue points are for the drug group. Figure (a) stands for China and (b) stands for the United States.
